# Schwerwiegende Late-onset-Neutropenien nach Rituximab-Gabe

**DOI:** 10.1007/s00393-025-01668-2

**Published:** 2025-06-04

**Authors:** Carla E. Schütte, Christina Hillebrecht, Jens G. Kuipers, Dirk O. Stichtenoth, Patrick-Pascal Strunz, Johannes Heck

**Affiliations:** 1 Institut für Klinische Pharmakologie, Medizinische Hochschule Hannover, Carl-Neuberg-Str. 1, 30625 Hannover, Deutschland; 2 Klinik für internistische Rheumatologie, Rotes Kreuz Krankenhaus, St.-Pauli-Deich 24, 28199 Bremen, Deutschland; 3 Medizinische Klinik II, Rheumatologie/Klinische Immunologie, Universitätsklinikum Würzburg, Oberdürrbacher Str. 6, 97080 Würzburg, Deutschland

**Keywords:** Anti-CD20-Antikörper, Unerwünschte Arzneimittelwirkung, Blutbildstörungen, G‑CSF, Rheumatologie, Anti-CD20 antibody, Adverse drug reaction, Blood dyscrasias, G‑CSF, Rheumatology

## Abstract

Rituximab (RTX) bewirkt durch Bindung an das CD20-Antigen eine B‑Zell-Depletion. Eine weniger bekannte unerwünschte Arzneimittelwirkung von RTX ist die ca. 6 Monate nach Therapiebeginn auftretende Late-onset-Neutropenie (LON). Als klinisches Management sollten unter RTX-Therapie regelmäßige Differenzialblutbildkontrollen erfolgen. Bei akuten LON-Episoden kann neben einer Watch-and-Wait-Strategie eine unterstützende Gabe von G‑CSF („granulocyte colony-stimulating factor“) diskutiert werden. Die Reexposition mit RTX nach stattgehabter LON wird nach Abwägung von Risikofaktoren und ausführlicher Aufklärung im Allgemeinen als vertretbar erachtet. In dieser Fallserie möchten wir 2 Fälle von rheumatologischen Patienten mit schwerwiegender LON nach RTX-Gabe vorstellen.

## Abstract (en)

Rituximab (RTX) causes B‑cell depletion by binding to the CD20 antigen. A less well-known adverse drug reaction of RTX is late-onset neutropenia (LON), which occurs approximately 6 months after treatment initiation. As clinical management, regular differential blood cell counts should be performed during RTX treatment. Besides a watch-and-wait strategy, supportive administration of granulocyte colony-stimulating factor (G-CSF) can be discussed for acute LON episodes. Re-exposure to RTX after LON has occurred is generally considered acceptable after diligent benefit-risk evaluation and thorough patient information. In this case series, we would like to present 2 cases of rheumatology patients with severe LON after RTX administration.

Rituximab (RTX) ist ein monoklonaler Antikörper, der durch Bindung an das CD20-Antigen eine Depletion von B‑Zellen innerhalb von 72 h bewirkt [2]. Die B‑Zell-Zahl erholt sich ohne erneute RTX-Exposition in den darauffolgenden 12 Monaten zumeist vollständig [2]. RTX ist bei rheumatoider Arthritis (RA) in Kombination mit Methotrexat (MTX) sowie bei ANCA-assoziierten Vaskulitiden in Kombination mit Glukokortikoiden zugelassen. Bei vielen weiteren rheumatischen Erkrankungen (z. B. Kollagenosen) wird es „off-label“ eingesetzt. Zu den typischen unerwünschten Arzneimittelwirkungen (UAW) zählen u. a. Infektionen [2].

Eine weniger bekannte unerwünschte Arzneimittelwirkung (UAW) von RTX ist die frühestens nach 4 Wochen, in der Mehrzahl der Fälle aber nach 5 bis 6 Monaten auftretende Late-onset-Neutropenie (LON) mit einer in der Regel selbstlimitierenden Reduktion der neutrophilen Granulozyten („absolute neutrophil count“ [ANC]) auf <1500/μl [2, 9, 16]. Es handelt sich bei der LON um eine Ausschlussdiagnose. Pathomechanistisch wird eine Fehlregulierung im Rahmen der B‑Zell-Rekonstitution angenommen, die mit einer überschießenden Produktion des „B-cell activating factor“ (BAFF) assoziiert ist. Dies könnte zu einer Förderung der B‑Zell-Lymphopoese zuungunsten der Granulopoese führen. Für die Entwicklung einer LON nach Gabe von RTX werden in der Literatur als prädiktive Faktoren hohe Ausgangswerte des proliferationsinduzierenden Liganden APRIL und gewisse Einzelnukleotidpolymorphismen des Fcγ-Rezeptor-Gens genannt [9]. Weitere Mechanismen könnten die Bildung antineutrophiler Antikörper sowie CD95-induzierte Apoptose durch große granulierte Lymphozyten sein [8].

## Falldarstellungen

### Fall 1

Ein 47-jähriger Patient wurde im August 2022 in die Klinik für internistische Rheumatologie des Roten Kreuz Krankenhauses Bremen aus einer chirurgischen Abteilung bei Zustand nach Fahrradsturz aufgrund einer im Behandlungsverlauf neu aufgetretenen progredienten Neutropenie übernommen. Begleitend wurde ein Herpes zoster festgestellt und mit Aciclovir therapiert. Als Grunderkrankung lag eine systemische Sklerose vor, welche zuletzt im April sowie im Mai 2022 (mit 14-tägigem Abstand) aufgrund einer neu aufgetretenen kardialen Beteiligung mit RTX behandelt worden war. Im Rahmen der systemischen Sklerose bestanden des Weiteren eine terminale Niereninsuffizienz mit Dialysepflicht, eine Polyarthritis, „puffy fingers“, Teleangiektasien im Gesicht und an den Handinnenflächen, eine verkleinerte Mundöffnung, ein verkürztes Zungenbändchen sowie eine progrediente Lungenbeteiligung mit pulmonalarterieller Hypertonie. Laborchemisch zeigte sich eine Leukozytopenie mit einer Leukozytenzahl von 800/µl mit ausgeprägter Neutropenie (ANC 100/µl). Während des Aufenthalts entwickelte der Patient eine Infektion eines einliegenden Demers-Katheters, welche antibiotisch behandelt wurde. Nach Ausschluss typischer klinischer Ursachen für eine Leukopenie wurde schließlich eine LON diagnostiziert. Im Verlauf erholten sich die Leukozyten- und Neutrophilenzahlen spontan. Aufgrund der schwerwiegenden Neutropenie erfolgte nach Entlassung die Umstellung auf Mycophenolat-Mofetil (MMF). Hierunter trat ein septischer Schock bei Dickdarmileus auf, welcher operativ versorgt werden musste. Bei stabiler Situation im Verlauf erfolgte eine Dosissteigerung von MMF ohne erneute Rituximab-Reexposition.

### Fall 2

Ein 77-jähriger Patient wurde im August 2022 in der Notaufnahme des Roten Kreuz Krankenhauses Bremen mit einer schweren Leukopenie (Leukozytenzahl 1300/µl, ANC 100/µl) und diffusem Unwohlsein ohne Infektzeichen vorstellig. Als Vorerkrankungen lagen neben einer Kleingefäßvaskulitis unklarer Genese (es bestand ein zeitlicher Zusammenhang zur zweiten COVID-19-Booster-Impfung) eine zuletzt mit RTX und MTX behandelte RA mit Lungenbefall vor. Die letzten RTX-Gaben waren im März und im April 2022 (mit 14-tägigem Abstand) erfolgt. Auffällig war klinisch eine Periimplantitis an einem Unterkieferfrontzahnimplantat. Das hierbei dezent erhöhte CRP fiel von 1,8 auf 0,7 mg/dl unter der zahnärztlich verordneten Therapie mit Amoxicillin.

In der Gesamtkonstellation nach Ausschluss aller Differenzialdiagnosen wurde von einer LON im Rahmen der RTX-Gabe ausgegangen. Nach spontaner Erholung der Neutrophilenzahl 5 Tage später erfolgte eine Therapieumstellung auf Leflunomid, worunter der Patient allerdings eine arterielle Hypertonie (> 150/85 mm Hg) entwickelte, sodass eine Reexposition mit RTX versucht wurde. Unter dieser Therapie zeigte sich im bis dato 30 Monate währenden Verlauf keine erneute Neutropenie.

## Diskussion

Die LON nach RTX-Gabe ist eine in der Regel selbstlimitierende und nicht schwerwiegende UAW [1, 10, 15]. Die vorgestellten Fälle stellen diesbezüglich Ausnahmen dar, da Schwerwiegendheitskriterien wie Vorstellung in der Notaufnahme mit Hospitalisierung (Fall 2) bzw. Verlängerung einer Hospitalisierung (Fall 1) erfüllt waren. Allgemein scheint die LON nicht mit einer klinisch signifikant erhöhten Mortalität bzw. Morbidität einherzugehen [15]. Die Inzidenz liegt im Bereich von 1,3 % [13] bis 29,9 % [11]. Diese Bandbreite könnte unter anderem auf heterogene Patient:innen-Populationen in Bezug auf Grunderkrankungen zurückzuführen sein [15, 17]. Das Risiko für eine LON ist u. a. mit folgenden Risikofaktoren assoziiert: IgG-Rezeptor-Polymorphismen, Therapie mit weiteren Immunsuppressiva (z. B. MTX) und erhöhtes Lebensalter [2]. Bei Arai et al. wird zusätzlich das LON-Risiko als proportional abhängig von der kumulativen RTX-Dosis angesehen [1]. In einer großen retrospektiven Kohortenstudie von Zonozi et al. mit 738 Patient:innen, die eine B‑Zell-depletierende Therapie im Rahmen einer Autoimmunerkrankung erhielten, war das Risiko für eine RTX-assoziierte LON deutlich erhöht bei gleichzeitiger Therapie mit Cyclophosphamid sowie bei Vorliegen eines systemischen Lupus erythematodes [17]. Interessanterweise wurden LON auch im Zusammenhang mit anderen Anti-CD20-Antikörpern wie Obinutuzumab, Ocrelizumab und Ofatumumab beschrieben [3, 8, 12, 14], sodass möglicherweise von einem Klasseneffekt auszugehen ist [14]. Da es sich bei der LON, wie oben erwähnt, um eine Ausschlussdiagnose handelt, ist es wichtig, mögliche Differenzialdiagnosen zu berücksichtigen (Tab. 1; [7]).

**Tab. 1:** Wichtige Differenzialdiagnosen für eine Neutropenie bei rheumatischen Erkrankungen im Erwachsenenalter. (Nach [7])

Genese	Beispiele	Maßnahmen
Medikamentös-toxisch	– Hochakut: Metamizol-induzierte Agranulozytose – Frühzeitig: DMARD-assoziiert (IL-6-Rezeptor-Antikörper, TNF-Hemmer, Januskinaseinhibitoren, Azathioprin) – Verzögert: Rituximab-induzierte Late-onset-Neutropenie	– Therapiestopp bzw. Therapieunterbrechung – Ggf. G‑CSF-Gabe
Infektiös	Häufige Infektionen mit Zytopenie: HIV, EBV, CMV, Tuberkulose, Malaria	– Infektiologische Diagnostik – Antiinfektive Therapie
Substratmangel	Vitamin B_12_, Folsäure	– Spiegelbestimmung – Substitution
Hämatoonkologisch	T‑LGL-Leukämie; Leukämien und Lymphome mit Knochenmarksinfiltration	– Differenzialblutbild – Durchflusszytometrie des peripheren Blutes – Knochenmarkpunktion – Ursächliche Behandlung
Genetisch	Benigne familiäre Neutropenie	– Familienanamnese – Abstammungserhebung – Meist gutartig
Pooling	Hyperspleniesyndrom	– Screening auf Lebererkrankungen – Therapie der portalen Hypertension

*CMV* Zytomegalievirus, *DMARD* „disease-modifying antirheumatic drug“, *EBV* Epstein-Barr-Virus, *G‑CSF* „granulocyte colony-stimulating factor“, *HIV* humanes Immundefizienzvirus, *IL* Interleukin, *T‑LGL* „T-cell large granular lymphocytic“, *TNF* Tumornekrosefaktor

Für die Behandlung der LON existieren keine etablierten Leitlinien. Arai et al. beschreiben die Indikation für regelmäßige Differenzialblutbildkontrollen auch nach Ausheilung der LON, da die Möglichkeit einer erneuten Neutropenie ohne erneute RTX-Gabe besteht [1]. Während der akuten Phase einer LON wird des Weiteren die Gabe von G‑CSF diskutiert. Ohne Gabe von G‑CSF variiert die Länge der LON zwischen 6 und 77 Tagen (bei LON Grad III, d. h. ANC 500–1000/µl) [1], sodass die Gabe von G‑CSF hier vermutlich nur minimale Effekte hat [1, 15]. Im Gegensatz dazu stehen Patient:innen mit LON Grad IV (ANC <500/µl) und erhöhtem Risiko für infektiöse Komplikationen. Arai et al. zeigten, dass eine sofortige Gabe von G‑CSF schwere Infektionen verhinderte [1]. Konträr dazu beschrieben Monaco et al. keine therapeutische Notwendigkeit, da das Outcome nicht verändert würde [10]. Abschließend ist die Notwendigkeit einer G‑CSF-Gabe nicht geklärt, wenngleich sie in der Praxis doch verhältnismäßig häufig durchgeführt zu werden scheint, z. B. bei 69 % der Patient:innen bei Zonozi et al. [17]. Vor einem unkritischen Einsatz von G‑CSF bei entzündlich rheumatischen Erkrankungen muss indes gewarnt werden, da hierunter Schübe der Grunderkrankung, insbesondere die Lunge betreffend, beschrieben sind [6]. Eine kritische Risiko-Nutzen-Abwägung ist in jedem Fall erforderlich.

Dem Risiko eines LON-Rezidivs stehen bei einer potenziellen Reexposition mit RTX dessen therapeutische Vorteile gegenüber. In mehreren Untersuchungen kam es bei Reexposition mit RTX zu keinem LON-Rezidiv [1, 4], in 3 weiteren Analysen fand sich dagegen ein Rezidivrisiko von 8 % [10], 15,8 % [13] bzw. 21 % [17]. Im Allgemeinen wird nach Abwägung von Risikofaktoren und Aufklärung die Reexposition mit RTX als vertretbar angesehen [10, 17]. Bei Reexposition mit RTX sollte auf Differenzialblutbildkontrollen in den 5 bis 6 Monaten nach RTX-Gabe geachtet werden [5], z. B. alle 4 Wochen.

Eine Analyse der hier vorgestellten Kasuistiken zeigt, dass das Vorgehen im zweiten Fall mit der bisherigen Studienlage konform ist. Es wurde eine spontane Ausheilung ohne therapeutische Unterstützung angestrebt. Nach Ausheilung der LON wurde sich nach initialer Änderung des therapeutischen Konzepts von RTX auf Leflunomid und nachfolgender Komplikation hierunter (arterielle Hypertonie) für eine Reexposition mit RTX entschieden. Die wiederholte Gabe von RTX kann als vertretbar angesehen werden und illustriert eine erfolgreiche Reexposition trotz schwerer LON.

Beim ersten Fall hätte aus theoretischen Überlegungen im Hinblick auf das Infektrisiko eine Therapie mit G‑CSF in Betracht gezogen werden können. Auch während der Infekte (VZV-Reaktivierung im Sinne eines Herpes zoster und Demers-Katheter-Infektion mit *Staphylococcus epidermidis*) hätte eine Therapie mit G‑CSF unter Umständen die Dauer der LON-Episode und eventuell das zweite Infektionsgeschehen verkürzen können. Retrospektiv betrachtet, hätte zusätzlich zu der G‑CSF-Gabe eine Reexposition mit RTX erwogen werden können, da sich bei dem Patienten unter der alternativ gewählten Therapie mit MMF Komplikationen ergaben (septischer Schock bei Dickdarmileus).

## Fazit für die Praxis

- Die Late-onset-Neutropenie (LON) als unerwünschte Arzneimittelwirkung nach Rituximab(RTX)-Gabe ist im Allgemeinen selbstlimitierend und verläuft in der Regel nicht schwerwiegend.
- Als klinisches Management sollten in den 5 bis 6 Monaten nach RTX-Gabe regelmäßige Differenzialblutbildkontrollen erfolgen, z. B. alle 4 Wochen.
- Bei einer akuten LON-Episode kann neben einem Abwarten der spontanen Ausheilung eine Verlaufsunterstützung mit G‑CSF („granulocyte colony-stimulating factor“) als individuelle Entscheidung in Abwesenheit konkreter Leitlinienempfehlungen diskutiert werden.
- Die Reexposition mit RTX wird nach Abwägen von Risikofaktoren und ausführlicher Aufklärung im Allgemeinen als vertretbar erachtet.

## Abkürzungen

ANC: Absolute neutrophil count
ANCA: Antineutrophile zytoplasmatische Antikörper
BAFF: B‑cell activating factor
CMV: Zytomegalievirus
CRP: C‑reaktives Protein
DMARD: Disease-modifying antirheumatic drug
EBV: Epstein-Barr-Virus
G‑CSF: Granulocyte colony-stimulating factor
HIV: Humanes Immundefizienzvirus
IL: Interleukin
LON: Late-onset-Neutropenie
MMF: Mycophenolat-Mofetil
MTX: Methotrexat
RA: Rheumatoide Arthritis
RTX: Rituximab
T‑LGL: T‑cell large granular lymphocytic
TNF: Tumornekrosefaktor
UAW: Unerwünschte Arzneimittelwirkung
VZV: Varizella-Zoster-Virus
